# Multifunctional electrospun nanofibrous scaffold enriched with alendronate and hydroxyapatite for balancing osteogenic and osteoclast activity to promote bone regeneration

**DOI:** 10.3389/fbioe.2023.1302594

**Published:** 2023-11-09

**Authors:** Shabnam Anjum, Dilip Kumar Arya, Mohammad Saeed, Daoud Ali, Mohammad Saud Athar, Wang Yulin, Saud Alarifi, Xixi Wu, P.S. Rajinikanth, Qiang Ao

**Affiliations:** ^1^ Department of Tissue Engineering, School of Intelligent Medicine, China Medical University, Shenyang, Liaoning, China; ^2^ NMPA Key Laboratory for Quality Research and Control of Tissue Regenerative Biomaterial, Institute of Regulatory Science for Medical Device, National Engineering Research Center for Biomaterials, Sichuan University, Chengdu, Sichuan, China; ^3^ Department of Pharmaceutical Sciences, Babasaheb Bhimrao Ambedkar University, Lucknow, India; ^4^ Department of Pharmacology, Dr. A.P.J. Abdul Kalam Technical University, Lucknow, India; ^5^ Department of Zoology, College of Science, King Saud University, Riyadh, Saudi Arabia; ^6^ Department of Chemistry, Aligarh Muslim University, Aligarh, India

**Keywords:** electrospinning, alendronate, hydroxyapatite nanoparticle, nanofibrous scaffold, bone regeneration

## Abstract

Electrospun composite nanofiber scaffolds are well known for their bone and tissue regeneration applications. This research is focused on the development of PVP and PVA nanofiber composite scaffolds enriched with hydroxyapatite (HA) nanoparticles and alendronate (ALN) using the electrospinning technique. The developed nanofiber scaffolds were investigated for their physicochemical as well as bone regeneration potential. The results obtained from particle size, zeta potential, SEM and EDX analysis of HA nanoparticles confirmed their successful fabrication. Further, SEM analysis verified nanofiber’s diameters within 200–250 nm, while EDX analysis confirmed the successful incorporation of HA and ALN into the scaffolds. XRD and TGA analysis revealed the amorphous and thermally stable nature of the nanofiber composite scaffolds. Contact angle, FTIR analysis, Swelling and biodegradability studies revealed the hydrophilicity, chemical compatibility, suitable water uptake capacity and increased *in-vitro* degradation making it appropriate for tissue regeneration. The addition of HA into nanofiber scaffolds enhanced the physiochemical properties. Additionally, hemolysis cell viability, cell adhesion and proliferation by SEM as well as confocal microscopy and live/dead assay results demonstrated the non-toxic and biocompatibility behavior of nanofiber scaffolds. Alkaline phosphatase (ALP) and tartrate-resistant acid phosphatase (TRAP) assays demonstrated osteoblast promotion and osteoclast inhibition, respectively. These findings suggest that developed HA and ALN-loaded PVP/PVA-ALN-HA nanofiber composite scaffolds hold significant promise for bone regeneration applications.

## Graphical Abstract

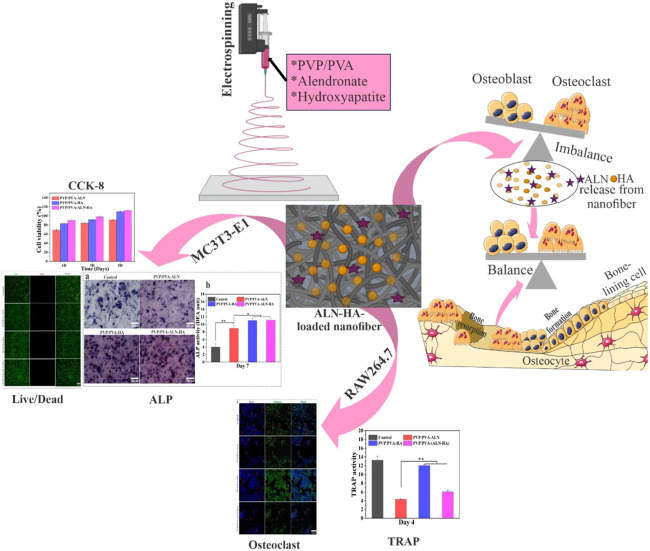

## 1 Introduction

The use of biomaterial-based bone grafts as alternatives for natural bone healing is common, and they have several benefits over previous grafting methods. The shattered bone underwent ineffective self-healing and slow auto-regeneration. Maintaining the immunological microenvironment’s regulation, balancing the interplay between bone formation and resorption, and establishing a functional neovascularization network are crucial factors. The formidable task lies in creating a remarkably bioactive scaffold for bone tissue engineering, capable of orchestrating the bone remodeling process to effectively enhance bone regeneration (Zhu et al., 2021). Bone remodeling constitutes an intricate biochemical response involving the osteoclast-driven bone resorption and osteoblast-mediated bone formation processes. It is important to highlight that several bone tissue regeneration frameworks have focused solely on stimulating the differentiation of bone stem cells to encourage bone reformation, often overlooking the interdependent interplay between bone resorption (osteoclasts) and bone formation (osteoblasts). Research prior to this has demonstrated that concurrently administering bone morphogenetic proteins (BMPs) and connective tissue growth factor (CTGF) remarkably augments the process of bone remodeling and accelerates the healing process (Cheng et al., 2019). Additionally, this approach predominantly emphasizes bone reformation through the stimulation of bone stem cell differentiation and the facilitation of angiogenesis. However, it tends to overlook the significant impact of osteoclastogenesis on the overall process of bone healing.

Nowadays, biomaterial-based bone grafts have gained extensive utilization as substitutes for bone healing. These grafts offer numerous advantages compared to alternatives developed using different grafting methodologies (Ahmed et al., 2021). Tissue engineering concepts have introduced fresh possibilities through the combination of suitable scaffolds, cells, and growth factors. These innovative strategies use scaffolds for regenerative purposes in the repair of damaged tissues (Anand et al., 2023). Extensive research has demonstrated that the electrospinning technique is a superb method for producing nanofibers characterized by finely controllable hierarchical structures and desirable constituents (Anand et al., 2022a; Thakur et al., 2023). The electrospun nanofibers possess a distinctive hierarchical micro/nanofiber architecture, which incorporates a highly porous structure. This configuration facilitates exceptional transmission of essential elements such as nutrients, oxygen, growth factors, and waste materials (Pandey et al., 2023; Singh et al., 2023). Their effectiveness in supporting bone regeneration hinges on their pivotal contributions to cell recruitment, cytokine release, and the transport of oxygen, nutrients, and metabolic waste. The absence of these functions would render successful bone regeneration unachievable (Jin et al., 2022; Sadeghi et al., 2022). The remarkable mechanical properties of nanofibers establish them as temporary mechanical barriers, effectively thwarting the undesirable migration of fibroblasts. Simultaneously, they promote the proliferation and differentiation of osteoblasts, crucial for the generation of new bone tissue.

Electrospun composites are crafted to harness the benefits of diverse materials within a single scaffold, aiming to capitalize on their combined advantages. Numerous polymer blends and composites have been successfully electrospun into unified mats through the utilization of both single and twin extrusion systems. Among these, PVA and PVP have excellent material properties in biomedical applications. PVA has exceptional features such as good biocompatibility, suitable degradability, good chemical resistance properties and hydrophilicity (Chahal et al., 2016). In addition, PVP is biocompatible and nontoxic, and has better blending abilities with other materials, to form composite materials. The Hydroxyl group of PVA and the proton accepting carboxyl groups of PVP intend to interact with each other to form a polymeric scaffold with good mechanical properties and controlled solubility (Chaudhuri et al., 2016). The fabricated composite scaffold consisting of bioactive polymers and ceramics will be further exposed to the differentiation and proliferation of osteoblasts for artificial bone formation.

HA, a predominant constituent of bones, stands as a biomaterial valued for its notable biocompatibility and osteoconductivity. Among ceramic bone grafts, HA is widely employed and serves as a suitable coating for orthopaedic implants but HA exhibits limitations in terms of its slow biodegradability and relatively modest mechanical properties. (Brannigan and Griffin, 2016; Anjum et al., 2022). Notably, combining HA with scaffolds has been shown to create an optimal microenvironment, closely mimicking the natural bone conditions (Balaji Raghavendran et al., 2014). Beyond this, HA particles have been explored as carriers for diverse drugs and proteins, including antibiotics and growth factors (Brannigan and Griffin, 2016).

ALN is a member of nitrogen containing bisphosphonate. It contains two side chains, one (-OH) is responsible for bone affinity and second one -(CH_2_)_3_-NH_2_ is responsible for antiresorptive capacity (Gong et al., 2011). It is used as an amino-bisphosphonate medication that may be useful in treating conditions including osteoporosis, Paget’s disease, hypercalcemia from malignancies, metastatic bone disease, and hypercalcemia from periodontal disease that are characterized by aberrant bone turnover (Balaji Raghavendran et al., 2014). ALN is found to be more potential and specific due to the presence of a primary amino group in the side chain (Palazzo et al., 2007). Many researchers have documented and substantiated the efficacy of ALN, both *in vitro* and *in vivo*, as a potent inhibitor of bone resorption. This effectiveness stems from its ability to suppress osteoclast activity (Drake, Clarke, and Khosla, 2008; Gong et al., 2011). However, due to its hydrophilic nature and intense polarity, ALN has low bioavailability in aqueous circumstances, which is one of its main drawbacks. An intriguing observation lies in ALN’s robust affinity for HA, opening the possibility of hybridizing the two to address the challenge of low bioavailability (Al-Baadani et al., 2022; Klara and Joanna, 2022). Furthermore, the calcium phosphate crystals present in HA exhibit a strong binding capability with ALN molecules, leading to an optimal loading of ALN. For instance, Shen et al. devised ALN-loaded HA titanium dioxide nanotubes, ensuring a controlled drug release that enhanced bone regeneration around implants in an osteoporosis model (Shi et al., 2009; Shen et al., 2016; Wang et al., 2019). In another investigation, Qu et al. successfully incorporated ALN into ultrahigh molecular weight polyethylene, achieving a sustained local release that effectively prevented osteolysis stemming from artificial joint wear (Yang et al., 2012). Assessing the osteogenic effects, Young et al. cultivated adipose-derived stem cells on ALN-loaded PCL nanofibrous scaffolds, revealing promising results with the 10% ALN/PCL scaffolds demonstrating significant alkaline phosphatase activity. These findings highlight the favourable impact of ALN/PCL nanofibrous scaffolds on bone regeneration (Yun et al., 2014).

In this research, the PVP/PVA nanofiber scaffolds loaded with ALN and HA was fabricated and characterized for its physicochemical properties, haemocompatibility, cytocompatibility (e.g., cell adhesion and proliferation on MC3T3-E1 bone cell line) and osteogenic and osteoclast activity.

## 2 Materials and methods

### 2.1 Materials

PVA (Mw: 80,000), PVP (Mw: 90,000), Alendronate sodium (Mw: 325.12), Calcium hydroxide Ca(OH)_2_ and Ortho-phosphoric acid (H_3_PO_4_) were purchased from Macklin Biochemical Co. Ltd. (P.R. China). Cell Counting Kit-8 purchased from KeyGEN BioTECH (China). MC3T3-E1 and RAW 264.7 cell lines were gift sample from professor Huang Zhongbing (Biomedical Engineering College; Sichuan University). All additional chemicals and reagents employed in the study were of analytical grade.

### 2.2 Synthesis of hydroxyapatite nanoparticle

The preparation of HA nanoparticles were carried out by the following procedure outlined in a previous reference (Ganachari et al., 2016). To elaborate briefly, aqueous solutions of 0.3 M H_3_PO_4_ and 0.5 M Ca(OH)_2_ were meticulously prepared. The H_3_PO_4_ solution was meticulously loaded into a burette, while the calcium hydroxide Ca(OH)_2_ solution was placed within a conical flask, ensuring thorough dissolution of all lime particles in water. The H_3_PO_4_ solution was then cautiously added drop by drop, maintaining a rate of approximately 2 mL/min, to the Ca(OH)_2_ solution. Throughout the process, the pH was gauged using a pH meter and upheld within a basic range through the introduction of ammonium hydroxide. The experimental conditions encompassed a temperature between 40°C and 45°C, pH between 8.5 and 10, and the solution was kept in continuous agitation. As the solution turned milky, it was left to undergo a 24 h ageing period, resulting in the formation of a precipitate. This precipitate was subsequently vacuum-dried at 60°C for an overnight duration. The experiment meticulously maintained a stoichiometric Ca/P ratio, while the basic medium was upheld for result comparison purposes.

### 2.3 Fabrication of PVP/PVA-ALN-HA nanofiber composite scaffold

The optimal concentrations for the electrospinning solutions have been detailed in Table 1. The PVP/PVA-ALN-HA nanofiber scaffold was prepared by the selection of optimum concentration of PVP/PVA nanofiber suitable for electrospinning. Initially, 12% PVP and 8% PVA solutions were made in 90% acetic acid and distilled water, respectively, under continuous stirring for 8 h, in a ratio of 50/50 to prepare homogenous blends for electrospinning. The electrospinning solution for PVP-PVA-ALN-HA was generated by dissolving 2% ALN and 2% HA, combined at a 1:1 ratio (ALN 200 mg and HA 200 mg), in 10 mL of 90% acetic acid through ultrasonic dispersion for 30 min. Subsequently, 1.2 g of PVP was introduced to this solution, and after 1.5 h, 10 mL of 8% PVA solution was incorporated into the PVP-ALN-HA mixture. The resultant solution was then vigorously stirred until it achieved transparency.

**TABLE 1:** Optimized concentration of polymers and drug solution for electrospinning.

Formulation	PVA (%)	PVP (%)	Ratio	HA	ALN
PVA-PVP-HA	8	12	50/50	2%	-
PVA-PVP-ALN	8	12	50/50	-	2%
PVA-PVP-ALN-HA	8	12	50/50	2%	2%

The formulated solutions were loaded into a 10 mL syringe outfitted with a stainless-steel needle featuring an inner diameter of 0.4 mm. A positive electrode was connected to the needle to administer high-voltage power. A flat collector, enveloped in aluminum foil, was utilized to gather the nanofibers and was placed 10 cm away from the spinning electrode. Regulating the electrospinning procedure, the flow rate of the syringe pump was calibrated to 2 mL/h, and the solutions were propelled using a robust electric voltage of 22 kV.

### 2.4 Physiochemical characterization

#### 2.4.1 Particle size distribution and zeta potential analysis

The particle size and zeta potential of the nanoparticles were assessed using a Malvern Instrument Zetasizer, nano-series, which employs laser Doppler anemometry and photon correlation spectroscopy. A quantity of 0.01 mg of the sample was introduced into 20 mL of water, and subsequently, 2 mL of the resulting solution underwent sonication. The sample was then moved to a cuvette for the determination of size and zeta potential at room temperature (Rajinikanth, Sankar, and Mishra, 2003; Al-Baadani et al., 2022; Deepak et al., 2023).

#### 2.4.2 Scanning electron microscopy (SEM) and energy dispersive X-ray spectroscopy (EDX) analysis

SEM (S4800; Hitachi Japan) was employed to characterize the morphology of the electrospun nanofiber batches. In this study, nanofiber samples were affixed onto SEM specimen stubs using double-sided carbon tape and subsequently coated with an Au-Pd layer via sputtering for 60 s. The analysis was conducted at varying magnifications and an accelerating voltage. For diameter determination, ImageJ software was employed, measuring the nanofiber scaffolds’ diameter at multiple locations. An EDS (S4800; Hitachi Japan) was also employed to analyse the elemental composition and distribution within the nanofiber scaffolds (Pandey et al., 2022; Yadav et al., 2022).

#### 2.4.3 Determination of λ_max_ of alendronate by UV–visible spectrophotometer

Spectroscopic analysis of alendronate was performed by a UV/Vis-spectrophotometer (U-3010; Hitachi Japan) in the UV range 200–800 nm for determining λ_max_. For this analysis, an alendronate sodium standard solution, (1 mg/mL) was prepared in distilled water. Aliquots of stock solution were transferred to a 10 mL of volumetric flask using a micropipette to give final concentration of 5, 10, 15, 20, 25, and 30 μg/mL. Since the ALN standard curve was unable to be established directly, the medium needed to be reacted with ninhydrin, as reported in previous literature [25]. 2.5 mL of 0.2% ninhydrin solution, 0.5 mL of 0.05 M sodium bicarbonate (NaHCO_3_) and the mixture was mixed well and heated in a water bath at 95°C ± 5°C for 35 min, resulting in purple solution. The flasks were cooled and the volume was made up to the mark with distilled water. The absorbance was measured at 568 nm against a reagent blank. Regression equation, correlation coefficient, slope and intercept were calculated from the graph.

#### 2.4.4 FTIR (fourier transform infrared spectroscopy)

FTIR (NEXUS 670; NICOLET, United States) was used to determine the constituting functional groups of different nanofibers. The structural changes occurring during electrospinning, blending, coating, etc. were scanned by FTIR, using standard KBr crystal at room temperature. The spectra were acquired in transmission mode spanning a wavenumber range from 4,000 to 400 cm^−1^, utilizing a resolution of 4 cm^−1^ (Anand et al., 2022b).

#### 2.4.5 XRD (X-ray diffraction)

The crystalline and amorphous characteristics of the synthesized nanomaterials and scaffolds were ascertained via X-ray diffractometer (DX-1000; PHILIPS United States). Samples were positioned on quartz zero background holders within a standard XRD setup. A solid-state Germanium detector cooled by liquid nitrogen was employed, and Cu K-alpha radiation at 45 kV and 40 mA current was utilized. Patterns were acquired across a 2θ range of 5°–80° at a scan rate of 2° per minute (Rajinikanth and Jestin, 2016).

#### 2.4.6 TGA (thermo-gravimetric analysis)

TGA (TGA/DSC 2/1600-ThermoStar; METTLER TOLEDO, Switzerland) was used to employed thermal stability of different nanofibers. All samples were heated at a rate of 10°C min^−1^ in a range of 25°C–800°C under a nitrogen atmosphere (Athar et al., 2023).

#### 2.4.7 Mechanical testing

The mechanical characteristics of the nanofibrous mats were assessed using a universal Testing Machine (UTM) (YG005A; Baien Instrument China) equipped with a 5 cN capacity load cell. Nanofibrous samples (*n* = 3) were cut into dimensions of 1 mm width and 20 mm length. These samples were positioned between two clamps and subjected to tensile displacement at a crosshead speed of 8 mm/min. From the stress-strain curve, the Young’s modulus, tensile strength, and elongation at break of the samples were determined.

#### 2.4.8 Hydrophilicity characterization by contact angle

The angle formed between the solid surface and the interface of the liquid or vapour is termed the contact angle (CA). The surface’s wetting properties, indicating its hydrophilicity or hydrophobicity, were assessed using a water contact angle instrument (JC 2000C1; POWEREACH China). To perform this evaluation, the sample was positioned on a flat surface, followed by carefully placing a water droplet onto it using a moving needle. The spherical image of the droplet was captured by a digital camera and projected onto a monitor, allowing for the measurement of the contact angle between the droplet and the nanofiber web surface. For data analysis, at least three measurements were taken at different locations on the film, and the values were averaged.

#### 2.4.9 *In vitro* drug release

To investigate drug release from the prepared nanofibers, the mats were cut into 1 × 1 cm^2^ sections and incubated in 10 mL of Phosphate buffer saline (PBS at pH 7.4). The incubation was conducted at 37°C with a shaking rate of 100 rpm in a thermostatic shaking incubator. Subsequently, 2 mL aliquots of the sample were withdrawn at predetermined time intervals (1, 2, 3, 4, 5, 6, 8, 10, 12 and 14 days), and equivalent volumes of fresh PBS were introduced to maintain a sink condition (Wu et al., 2014). The release of alendronate from different nanofiber groups was quantified using UV-visible spectroscopy, following a procedure outlined in another study (Gao et al., 2016). In essence, 1 mL of the release medium was combined with 2.5 mL of a 0.2% ninhydrin solution and 0.5 mL of 0.05 M NaHCO_3_. This mixture was thoroughly mixed and heated in a water bath at 95°C ± 5°C for 35 min, resulting in a purple solution of 4 mL. Finally, the purple solution was diluted with deionized water to a final volume of 5 mL, and the absorbance at 568 nm was measured.

#### 2.4.10 *In vitro* degradation

The degradation pattern of the nanofibers was examined by observing the weight loss at various time intervals. Scaffolds measuring 1 × 1 cm^2^ were meticulously weighed (W_i_) and then placed within 5 mL plastic tubes, each containing 4 mL of PBS solution with a pH of 7.4. These tubes were subsequently positioned in a shaking incubator at a speed of 100 rpm and a temperature of 37°C. The incubation media were changed on a weekly basis. At predetermined time points, the samples were subjected to drying until a consistent weight was achieved (W_f_) (Agarwal et al., 2021). The weight loss percentage (%) was determined using the formula:

$$Weight\;loss\;\left(\%\right)=\frac{W_{i}-W_{f}}{W_{i}}x100$$

Where *W*_*i*_ = initial weight of the sample and *W*_*f*_ = final weight after degradation.

#### 2.4.11 Water uptake capacity

The water retention ability of the scaffolds was assessed through the calculation of the swelling ratio. Initially, the samples were cut into dimensions of 1 × 1 cm^2^, and their dry weight (W_d_) was determined using an electronic weighing balance and duly recorded. Subsequently, these samples were immersed in PBS with a pH of 7.4 at room temperature. At specified intervals, the samples were taken out of the solution and placed on tissue paper to eliminate any excess water clinging to the nanofiber surface. The wet weight of the nanofibers (W_w_) was promptly measured. All measurements were conducted in triplicate, and the water uptake capacity was calculated using the following equation:

$$\%\text{Water uptake capacity}=\frac{W_{w}-W_{d}}{W_{d}}x100$$

Where *W*_*w*_ = weight of the wet nanofiber scaffold and *W*_*d*_ = dried weight.

A triplicate study was performed, and the average value was taken as the percentage of water uptake.

### 2.5 Hemolysis study

To determine the hemocompatibility of a developed PVP/PVA nanofiber scaffold loaded with ALN and HA encompassed the utilization of freshly obtained blood samples from healthy rats. Blood is a gift from professor Huang Zhongbing, (Biomedical Engineering College; Sichuan University). Blood specimens (5 mL each) were collected into heparin-coated tubes and subsequently subjected to centrifugation at 3,000 rpm, resulting in the separation of plasma from the red blood cells (RBCs) pellets settled at the bottom of the tubes. Careful removal of the liquid above the sediment was followed by the introduction of PBS into the tubes. Another round of centrifugation was employed to isolate the re-suspended RBCs. The resultant refined blood cells were diluted to attain a final volume of 25 mL, thus creating an RBCs suspension in PBS. Subsequently, 0.5 mL of this RBC suspension was divided into five 1.5 mL tubes. Within this set, two tubes were marked as negative control (PBS) with a pH of 7.4 and positive controls (water), received 1 mL, respectively. The remaining three tubes underwent treatment with nanofibers (PVP/PVA-ALN, PVP/PVA-HA and PVP/PVA-ALN-HA) sized at 1 × 1 cm^2^. All the tubes, encompassing both control and treated samples, were incubated at 37°C for a duration of 3 h. Following this incubation period, all tubes were centrifuged at 3,000 rpm for 10 min. After the incubation and centrifugation steps, 200 µL of the supernatant from each sample was transferred into a 96-well plate, enabling the measurement of haemoglobin absorption at a wavelength of 540 nm.

### 2.6 *In vitro* biological evaluation

#### 2.6.1 Scaffold sterilization

Prior to the cell-seeding process, it is crucial to sterilize the nanofibers. The procedure involved submerging the samples in 70% ethanol and subjecting them to ultravoilet (UV) light exposure on both sides for a duration of 30 min. Following this, the ethanol was eliminated, and the samples were rinsed with PBS three times before initiating the cell culture experiments.

#### 2.6.2 Cell culture and seeding

MC3T3-E1 cells were maintained in α-MEM (Gibco; United States) containing 10% foetal bovine serum (Amresco; United States) and 1% penicillin–streptomycin (Gibco; United States), and incubated at 37°C under a 5% CO_2_ atmosphere.

#### 2.6.3 *In vitro* cytotoxicity (CCK-8 assay)

Cell proliferation and viability of MC3T3-E1 cells were assessed using the Cell Counting Kit-8 in accordance with the manufacturer’s instructions. In triplicate, MC3T3-E1 cells (1 × 10^4^ cells/well) were seeded onto various nanofibers. Following cell culture in 96-well plates for 1, 3, and 5 days, a CCK-8 solution was introduced to each well. After a 2 h incubation at 37°C, the optical density (OD) at 450 nm was measured using a microplate reader (Thermo Fisher). Cell viability was determined using the following formula:

$$\mathrm{Cell viability}\left(\%\right)=\frac{{OD}_{Scaffold}}{{OD}_{Control}}\;x\;100\%$$

#### 2.6.4 Live/dead staining

To further determine the cytocompatibility of the fabricated scaffolds, MC3T3-E1 cells (1 × 10^4^ cells/well) were cultured for 3 days on the scaffolds. After incubation, the samples were washed thrice with PBS and stained with acetoxymethyl ester of calcein (calcein AM) and propidium iodide (PI) staining solution (Solarbio; China) for 20 min in the dark. Next, images were captured using by Inverted fluorescence microscope (Nikon).

#### 2.6.5 Cell adhesion and morphology

The investigation of cell adhesion was conducted using MC3T3-E1 cells cultured on PVP/PVA nanofiber scaffolds incorporating ALN and HA. Cell adhesion was assessed through the examination of adhered cell morphology. Nanofiber samples measuring 1 × 1 cm^2^ in diameter were positioned in 48-well culture plates. MC3T3-E1 cells (1 × 10^4^ cells/well) were cultured on fabricated scaffolds for 1 and 3 days. Following the designated incubation period, the culture medium was aspirated, and the cells were gently rinsed 3 times with PBS to remove any non-adherent cells. Subsequently, the adherent cells were fixed with a solution of 4% paraformaldehyde for 15 min at room temperature followed by permeabilization with 0.1% Triton X-100 for 5 min. Finally, the adhered cells were then air-dried, sputter-coated with gold or platinum, and the morphology was analysed by SEM.

The cell adhesion was also analyzed by visualizing the morphology of the adhered cells by staining the actin cytoskeleton structures. After the cell fixation, the scaffolds were stained with DAPI (Solarbio; China) and phalloidin (Solarbio; China) for 10 min and 1 h, respectively, at room temperature. The images were captured using confocal laser scanning microscopy (CLSM) (LSM880 Airyscan with STEDYCON; Carl Zeiss Germany) with 405 nm (blue, DAPI) and 561 nm (green, phalloidin) excitation filters.

#### 2.6.6 Alkaline phosphatase (ALP) activity

To assess the early osteogenic differentiation potential of MC3T3-E1 cells, ALP activity was carried out [16]. In 48-well plates, MC3T3-E1 cells at a density of 1 × 10^4^ cells per well were cultured on various nanofibers for 7 days. After the addition of a 1% Triton X-100 (Solarbio; China) solution, proteins were extracted. The resulting cell lysates underwent centrifugation at 12,000 rpm for 10 min, yielding supernatant for the ALP test. The Alkaline Phosphatase Assay Kit (Beyotime; China) was employed to measure ALP activity. The optical density (OD) values at 520 nm were determined using a spectrophotometric microplate reader.

For ALP staining, the adherent cells were fixed using 4% paraformaldehyde (Solarbio; China) and then subjected to staining using a BICP/NBP ALP Stain Development Kit following the manufacturer’s instructions (Beyotime; China). Representative images were captured using an inverted fluorescence microscope (Nikon).

#### 2.6.7 Osteoclast formation

RAW 264.7 cells (3 × 10^4^ cells/cm^2^) were seeded onto different nanofiber membranes in 48-well plates and cultured in DMEM (high glucose) medium supplemented with 10% foetal bovine serum (FBS). This medium was enriched with 50 ng/mL RANKL and 20 ng/mL MCSF for a period of 4 days. Subsequently, the cytoskeleton and nuclei of the adherent cells were fixed and stained using phalloidin and DAPI, respectively. The stained cells were examined using CLSM.

Additionally, the TRAP activity of the RAW 264.7 cells was evaluated using a TRAP Assay Kit (Beyotime, China) following the manufacturer’s instructions. The assessment was conducted using a spectrophotometric microplate reader, measuring the absorbance at 405 nm (Gong et al., 2011).

### 2.7 Statistical analysis

The experimental data in this study were presented as Mean ± SD, with a sample size of *n* = 3. Data analysis included the use of a one-way ANOVA followed by the Bonferroni multiple comparison test, conducted using OriginPro 2019b software. Statistical significance was indicated by a *p*-value of less than 0.05, denoted as *, while *p*-values less than 0.01 were indicated as **, representing high significance. In cases where the *p*-value was less than 0.001, the significance was considered very high and denoted as ***.

## 3 Result and discussion

### 3.1 Particle size distribution and zeta potential analysis of HA nanoparticle

**FIGURE 1:**
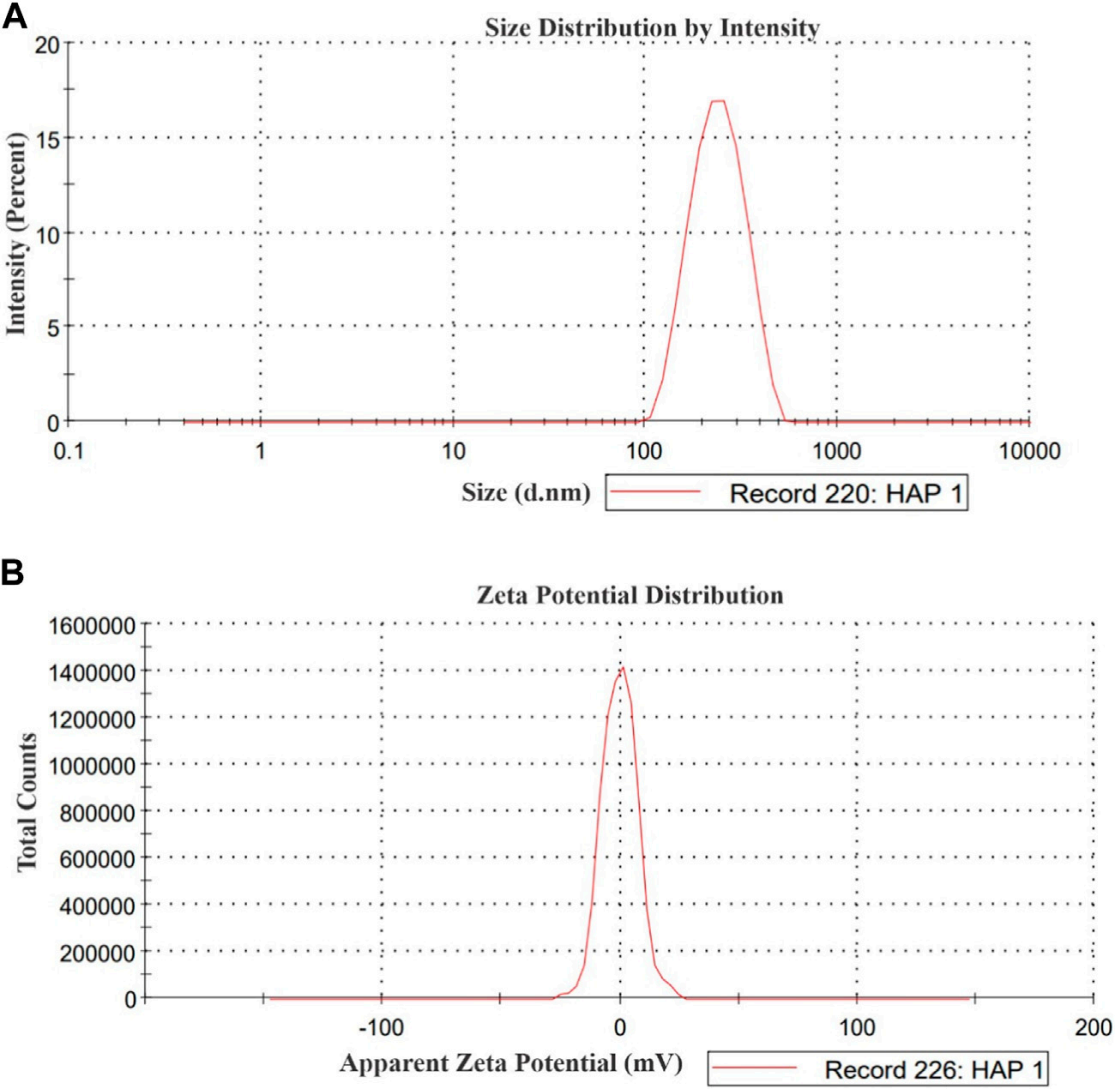
**(A)** Particle size distribution and **(B)** Zeta potential of HA nanoparticle.

The particle size distribution and zeta potential analysis of the HA sample is presented in Figure 1. The analysis revealed a wide peak of sizes spanning from 100 to 300 nm, accompanied by a polydispersity index (PDI) of 0.306. The average size of the HA particles was measured at 241.2 nm. The zeta potential of the HA sample was determined to be −0.11 mV.

### 3.2 Morphology of nanofibers and HA

**FIGURE 2:**
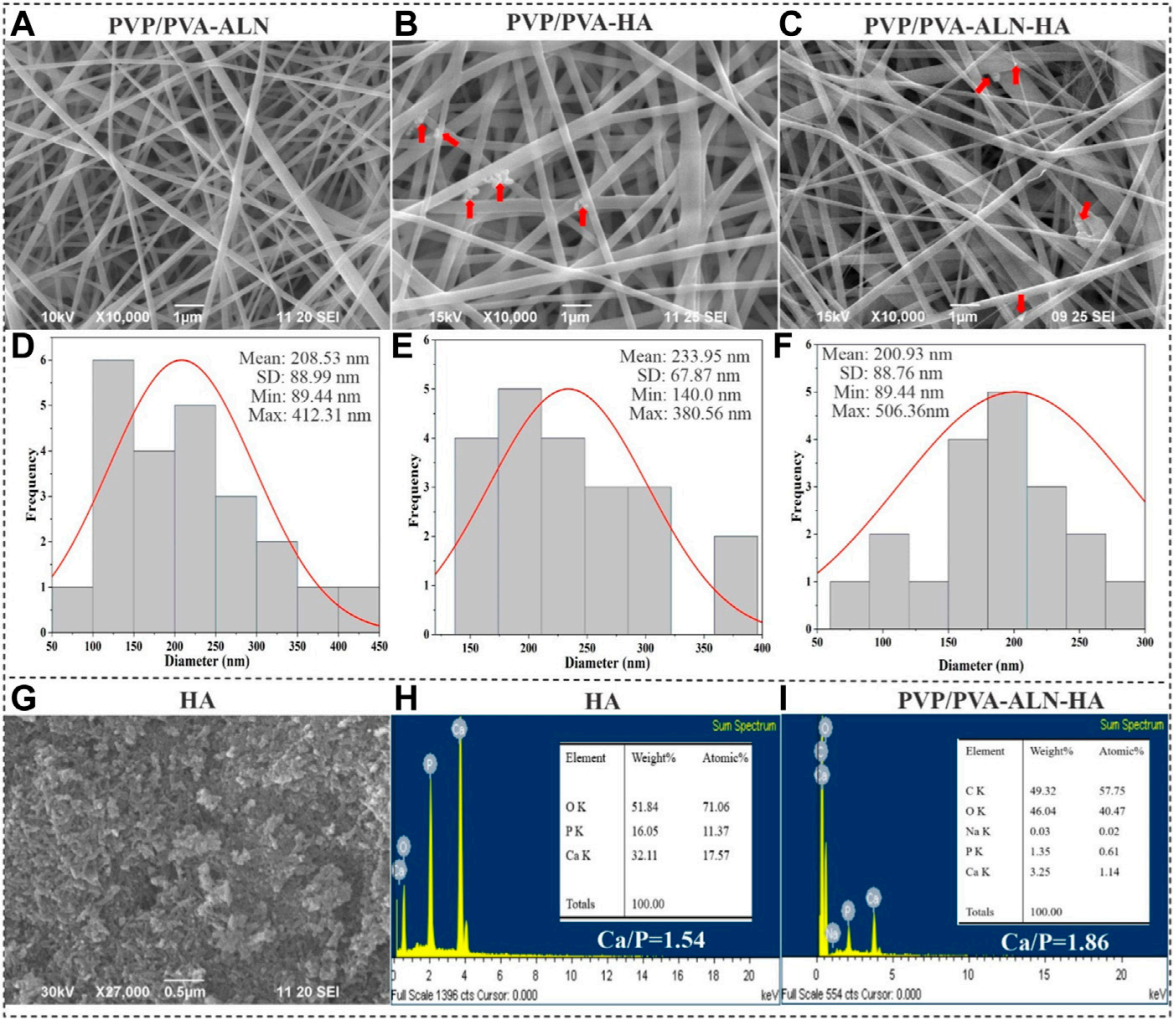
SEM images of **(A)** PVP/PVA-ALN, **(B)** PVP/PVA-HA and **(C)** PVP/PVA-ALN-HA nanofibers, and the red arrow in the SEM images indicates the presence of HA into nanofiber scaffolds; **(D–F)** Represents their corresponding histogram showing average diameters; **(G)** SEM image of HA nanoparticles; **(H)** EDX analysis of HA nanoparticles; **(I)** EDX analysis of PVP/PVA-ALN-HA nanofiber composite scaffold.

The morphological assessment of nanofibers, encompassing characteristics such as fibre shape, diameter, and surface structure, was carried out through SEM. This analysis unveiled the topographical features of nanofibers, a crucial factor influencing initial cell interactions like adhesion and proliferation (Teixeira et al., 2004). To explore the morphology of both pure HA and the composite nanofibers, the prepared scaffolds were subjected to SEM analysis (Figure 2). SEM images of PVP/PVA-ALN, PVP/PVA-HA, and PVP/PVA-ALN-HA showcased uniform nanofiber sizes that were randomly distributed, devoid of any bead defects (Figures 2A–C).

ALN and HA nanoparticles were successfully incorporated homogeneously into the nanofibers evidenced by SEM images. Additionally, HA particles were integrated within the structure of blend nanofiber scaffolds and appeared to have a rougher surface compared to PVP/PVA-ALN fibers, indicating the successful incorporation of HA into nanofibers. Visible aggregation of ALN-HA or HA can be observed within the nanofibers, as indicated by the red arrows as shown in Figures 2B, C. The rough surface of HA loaded nanofiber may be beneficial for cell attachment and proliferation.

The average diameters of the PVP/PVA-ALN, PVP/PVA-HA, and PVP/PVA-ALN-HA composite nanofibers were measured to be 208.53, 233.95, and 200.93 nm, respectively (Figures 2D–F). The data indicated that there was no notable distinction in nanofiber size between PVP/PVA-ALN, PVP/PVA-HA and PVP/PVA-ALN-HA. The SEM findings for the synthesized HA nanoparticles indicated grain sizes spanning from 50 to 250 nm, as displayed in Figure 2G. EDX results showed that HA nanoparticles had a Ca/P ratio of 1.55, very close to natural bone (Figure 2H). Further confirmation of the presence of HA and ALN within the PVP/PVA-ALN-HA nanofibers was attained through EDX spectra analysis (Figure 2I). The peaks attributed to sodium (Na) and phosphorus (P) could be attributed to the presence of both ALN and HA, while the calcium (Ca) peak indicated the integration of HA particles within the PVP/PVA nanofiber. Elemental analysis resulted in a Ca/P ratio of 1.86, closely resembling the stoichiometric ratio found in HA and approaching the ratio of 1.67 observed in natural bone tissue. This finding verifies the successful incorporation of ALN and HA within the nanofiber structure.

The nanofibrous scaffold, produced through the electrospinning process in this study, exhibited uniform orientation, interconnecting pores, and a web-like porous structure. This structural feature was crucial for facilitating oxygen exchange, fluid flow, nutrient transport, fibroblast infiltration, cell adhesion, and attachment. The outcomes demonstrated that the hybrid nanofibers developed possessed the desired physical and structural attributes necessary for bone tissue regeneration (BTR).

### 3.3 Physiochemical properties

#### 3.3.1 λ_max_ validation of ALN

**FIGURE 3:**
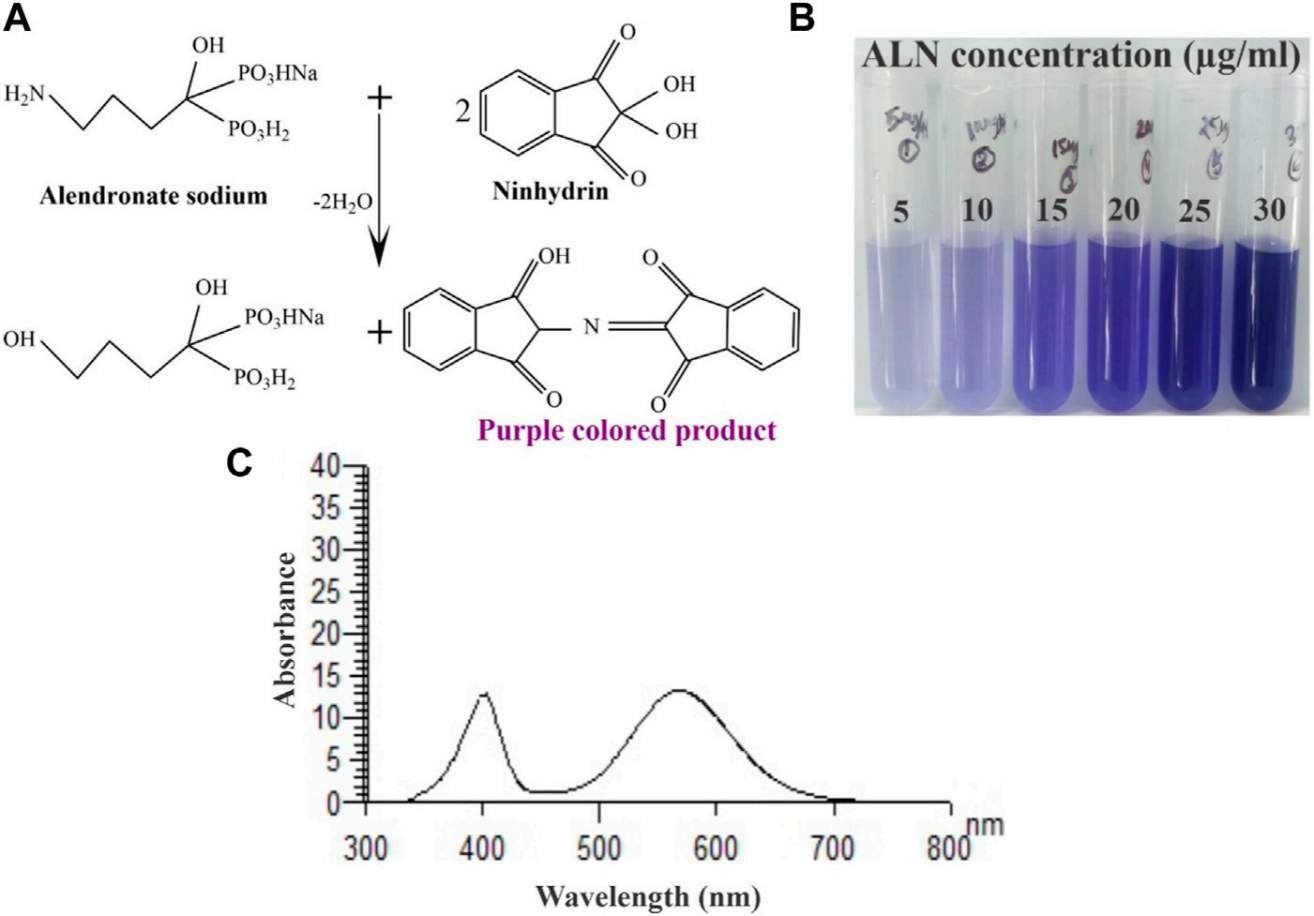
**(A)** Schematic representation of ninhydrin binding to ALN, **(B)** An image of the final reacted solutions which correspond to different ALN concentrations and **(C)** Absorption spectrum of the reaction product of alendronate sodium (25 μg/mL) with ninhydrin.

Alendronate contains a primary aliphatic amino group which is known to react with ninhydrin reagent. This reagent is used for the determination of primary amines and amino acids. UV-vis absorption spectrum of the purple-colour complex produced by the reaction of ninhydrin with alendronate was recorded in the range from 200 to 800 nm against reagent blank. The maximum absorbance λmax was found at 568 nm (Figures 3A–C). The reaction between ninhydrin and alendronate is temperature dependent.

#### 3.3.2 FTIR

**FIGURE 4:**
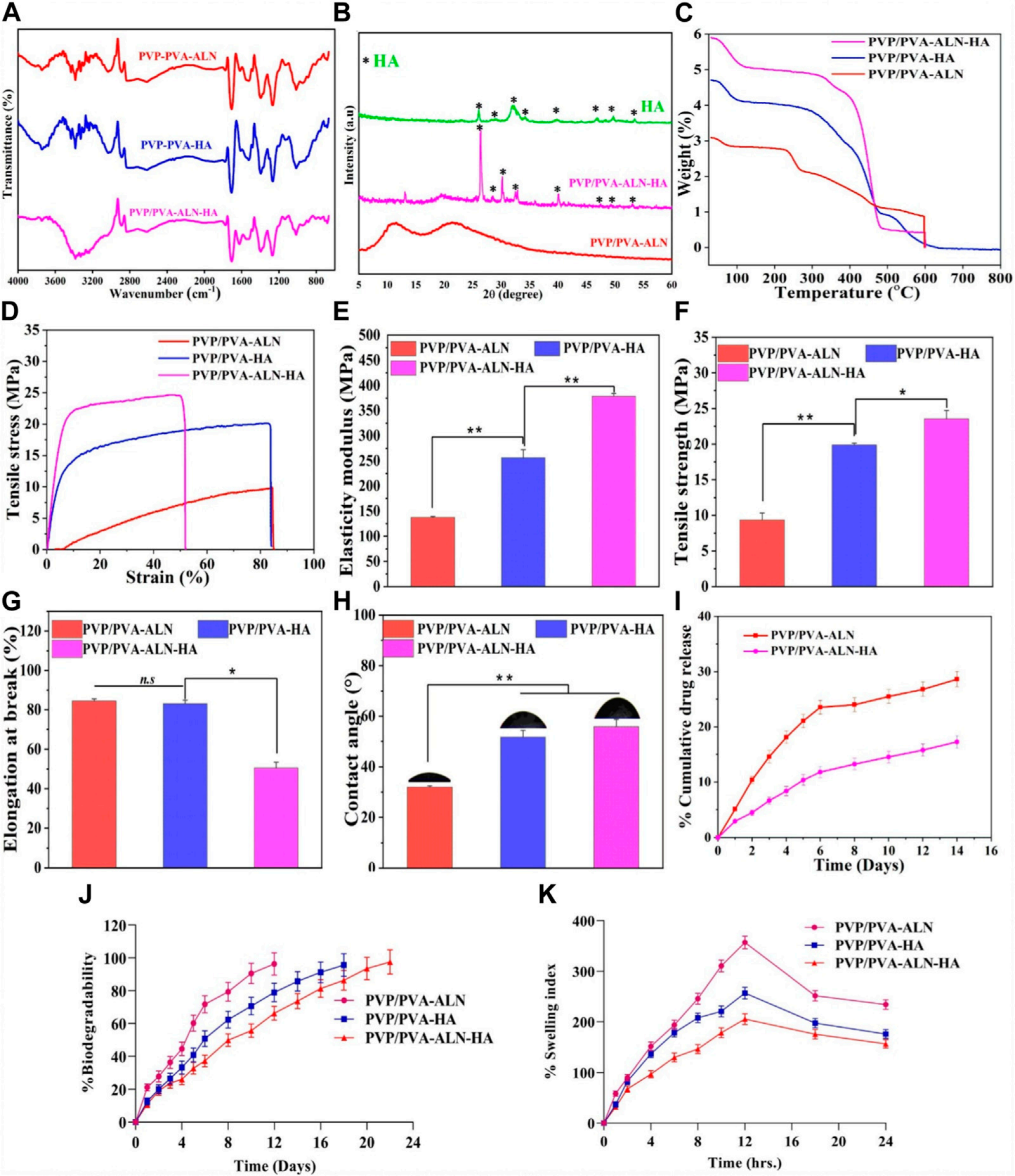
Different physiochemical properties of developed PVP/PVA-ALN, PVP/PVA-HA and PVP/PVA-ALN-HA nanofiber composite scaffolds; **(A)** FTIR spectra, **(B)** XRD spectra, **(C)** TGA analysis plot**, (D)** Stress *versus* strain plot, **(E)** histogram representing elasticity modulus in (MPa) **(F)** histogram showing the tensile strength graph in (MPa), **(G)** shows the elongation at break (%), **(H)** contact angle measurement, **(I)** %Cumulative drug release of Alendronate, **(J)** *in-vitro* biodegradability for 2 weeks and **(K)** swelling index (%) up to 24 h for nanofiber scaffolds.

The FT-IR spectrum shows prominent bands of HA along with the bands of PVP/PVA in the fabricated nanofibers (Figure 4A). The FTIR data of the nanofiber demonstrated a broad peak at 3,748 cm^−1^ along with strong intensity due to the stretching vibrations of hydroxyl group in PVP/PVA-ALN and PVP/PVA-HA nanofibers. But the intensity of peak of hydroxyl group in PVP/PVA-ALN-HA was less around 3,378 cm^−1^, it may be due to the hybridization of ALN and HA. The band at 1,016 cm^−1^ confirm the presence of C-O vibration of PVA-PVP (Salim et al., 2021). In addition, the presence of a 1,530 cm^−1^ peak was due to stretching vibrations of the carbonyl group present in PVA. The band at about 1,256 cm^−1^ corresponds to C-O stretching of acetyl groups present on the PVA backbone. The appearance of C-O stretching is due to the semi-crystalline nature of the blends. A band at 1,396 cm^−1^ is attributed to C-N bond, mainly from the functional group of PVP. The vibration band at about 1701 cm^−1^ corresponds to C-O symmetric bending of PVA and PVP. The FTIR spectra of the nanofiber blend agreed well with the reported values [30,32]. When ALN was added, no additional peaks of ALN were detected in these scaffolds may be due to the spectra of ALN and HA overlap. The characteristic peaks of crystalline phosphate (PO_4_^3−^) at 1,040, 920 and 805 cm^−1^ were detected in the FTIR spectra, confirming the presence of HA.

#### 3.3.3 XRD analysis

XRD analysis was conducted to explore the structural and crystalline properties of the prepared nanofibers (Figure 4B). The distinct peaks present in the pattern affirm the crystalline nature of the HA component. Conversely, the diffraction patterns of spectra showing broad halos indicate the amorphous nature of the polymers. The XRD spectrum for pure PVA/PVP demonstrates diffused and broadened peaks within the range of 2θ = 11.08°–21.26°, indicative of the amorphous nature of the polymers (Maheshwari et al., 2017).

HA features a crystalline structure with multiple diffractive planes, each manifesting its unique diffraction peak at a specific position. Various diffractive planes such as 002, 211, 202, 310, 222, and 213 are identified by sharp peaks at 26.1°, 32.1°, 34.2°, 39.9°, 46.7°, and 49.30°, respectively. This pattern strongly suggests the crystalline nature of the synthesized HA. In the case of composite scaffolds, the HA phase peak is noticeable (marked by *). In the context of polymer composites, the incorporation of ALN and HA results in weakened peak intensities for PVP/PVA-ALN-HA, without altering the peak positions. This phenomenon could be attributed to the enhanced diffraction of ceramic crystals or possibly an overlap of diffraction peaks from HA and ALN.

#### 3.3.4 TGA analysis

Thermal stability analysis of PVP/PVA-ALN, PVP/PVA-HA, and PVP/PVA-ALN-HA nanofibers was conducted using TGA, as depicted in Figure 4C. The figure illustrates the improved thermal stability of the polymer composite achieved through the incorporation of nanomaterials (such as HA) within the polymer matrix. The findings indicated that the weight loss occurring within the temperature range of 70°C–130°C was attributed to the evaporation of water content present in the samples. In PVP/PVA-HA, it can be seen from Figure 4C that the weight loss in two states at ∼370°C and 480°C, which was due to the loss of polymeric chain in the material and the entire material gets decomposed at 600°C which represents the remarkable thermal stability. PVP/PVA-ALN and PVP/PVA-ALN-HA showed the weight loss observed at ∼280°C, 460°C and 360°C, 485°C and total weight loss observed at ∼600°C, which might be due to the diminishing labile group and eruption of adhered material. Hence, these results revealed that the material is thermally stable.

#### 3.3.5 Mechanical strength measurement

The response of the developed nanofiber scaffolds to mechanical loads is presented in Figure 4D. The characteristic tensile stress-strain curves and corresponding tensile properties, including Young’s modulus, elongation-at-break, and tensile strength, are displayed (Figures 4D–G). The outcomes highlighted that all scaffolds exhibited a certain degree of stiffness and resistance to deformation. Previous research exhibited that the elastic modulus and tensile strength of human cancellous bone ranges from 0.05 to 0.5 GPa and 1–20 MPa, depending on the apparent density (Roeder et al., 2003). Comparing the PVP/PVA-ALN sample, the tensile strength of PVP/PVA-HA and PVP/PVA-ALN-HA displayed substantial enhancements upon the introduction of HA. Figure 4E revealed that the Young’s modulus of PVP/PVA-HA (255.94 MPa) and PVP/PVA-ALN-HA (372.32 MPa) exhibited the highest values compared to PVP/PVA-ALN (137.80 MPa). The maximum tensile strength (TS) of PVP/PVA-ALN-HA is evident in Figure 4F. The incorporation of HA led to improved mechanical properties of the nanofibers.

Furthermore, PVP/PVA-ALN exhibited TS of 9.23 MPa and an elongation at break (EB) value of 84.23% (Figure 4G). In contrast, PVP/PVA-HA and PVP/PVA-ALN-HA demonstrated TS and EB values of 19.90 MPa, 23.47 MPa, and 82.91%, 50.39%, respectively. It is worth noting that high porosity can negatively impact mechanical behaviour [28]. The lower TS of PVP/PVA-ALN compared to PVP/PVA-ALN-HA could be attributed to the limited bioavailability of ALN in aqueous conditions. The incorporation of ALN-HA nanoparticles resulted in an increased TS. Research has indicated that the addition of HA to electrospun blends enhances the mechanical properties of the nanofibers (Wuriantika et al., 2021).

In summary, the homogeneous distribution of ALN-incorporated HA nanoparticles along the fibre axis played a significant role in altering the mechanical properties of the nanofibers, enhancing their stiffness rather than flexibility. These results imply that PVP/PVA-ALN-HA holds promise for BTR. These enhancements in mechanical properties could effectively bolster the nanofiber’s ability to withstand external forces during surgical procedures, meeting the demands of functioning as a mechanical barrier.

#### 3.3.6 Contact angle

We conducted CA measurements on the scaffold to assess the influence of HA and ALN (Figure 4H). The average CAs for PVP/PVA-ALN, PVP/PVA-HA, and PVP/PVA-ALN-HA were found to be 31.75°, 51.69°, and 55.85°, respectively. The nanofiber loaded with ALN exhibited inherent hydrophilicity, while those loaded with HA displayed slight reduction in hydrophilic characteristics. Wettability stands as a crucial parameter for scaffolds, significantly impacting their mechanical stability, cell adhesion, and proliferation properties. It is noted that nanofiber surfaces serve as excellent platforms for sustained drug delivery, as they slow down degradation and extend the time for cell attachment prior to degradation, enhancing cell adhesion capabilities. This property holds immense importance for the application of nanofibers in BTR. In essence, the prepared nanofiber batches displayed appropriate contact angles, aligning with the requirements for an ideal scaffold in the context of BTR.

#### 3.3.7 Drug release behavior

The cumulative concentration of released ALN from PVP/PVA-ALN and PVP/PVA-ALN-HA is presented in Figure 4I. The results clearly illustrate that ALN was released at a slower rate from PVP/PVA-ALN-HA compared to PVP/PVA-ALN. This suggests that encapsulating ALN within HA nanoparticles can effectively extend the duration of drug release. It is noteworthy that both PVP/PVA-ALN and PVP/PVA-ALN-HA nanofibers exhibited an initial burst release in the first 3 days. However, the ALN from the PVP/PVA-ALN-HA nanofiber exhibited a more prolonged release pattern as compared to PVP/PVA-ALN, specifically observed between day 3 and day 14. Due to the potential for severe side effects associated with excessive ALN release, the controlled release of ALN from the nanofibers offers a way to mitigate the toxicity linked to high doses. Consequently, the utilization of PVP/PVA-ALN-HA nanofibrous scaffolds has the potential to facilitate extended ALN delivery and support the BTR process for targeted size defects.

#### 3.3.8 *In vitro* biodegradation

The degradation behavior of the scaffolds during the incubation period is depicted in Figure 4J. The results showed that nanofibers PVP/PVA-ALN had degraded by almost 96.35% after day 12 while PVP/PVA-HA showed a slower degradation rate 95.62% after 18 days. After addition of HA into nanofiber scaffold, PVP/PVA-ALN-HA nanofiber degraded more slowly and demonstrated 97.53% after 22 days. The variation in nanofiber degradation rates is directly attributed to the hydrophilic nature of the two polymers; higher hydrophilicity leads to faster degradation. This implies that the nanofibers would naturally dissolve within the body without necessitating their removal.

#### 3.3.9 % water uptake capacity

The swelling behaviour of the scaffold demonstrates its capacity to facilitate the exchange of nutrients and waste materials between the environment and the cells encapsulated within the scaffold, creating an environment conducive to artificial tissue production. Swelling refers to the scaffold’s ability to hydrate and stabilize within biological systems. It can be employed as a carrier material for facilitating cell proliferation and differentiation processes. Hydrophilicity is a crucial characteristic in tissue engineering scaffolds, as it can enhance cell viability and proliferation.

Significant disparities were observed among the three tested scaffold groups (PVP/PVA-ALN, PVP/PVA-HA, PVP/PVA-ALN-HA), as displayed in Figure 4K. Notably, PVP/PVA-ALN exhibited a remarkably high swelling ratio of approximately 356.92% after 12 h, surpassing PVP/PVA-HA at around 256.92% after 12 h of swelling index. This divergence might be attributed to the presence of ALN in PVP/PVA-ALN, which features hydrophilic groups, such as amino groups, enabling the penetration of water molecules within the scaffold’s chains. Consequently, this scaffold showcased the highest hydrophilicity and swelling capability when compared to the other scaffolds tested.

Upon incorporating HA nanoparticles into the nanofibers, a notable reduction in swelling capacity was observed. Specifically, PVP/PVA-ALN-HA nanofibers demonstrated a swelling ratio of approximately 205.24% after 12 h of swelling. This reduction implies that the inclusion of HA nanoparticles into the nanofibers significantly curtailed the swelling rate. This outcome can be attributed to the hybridization of ALN and HA nanoparticles, which collectively circumvent low bioavailability, resulting in a reduction in swelling.

### 3.4 Hemolysis study

**FIGURE 5:**
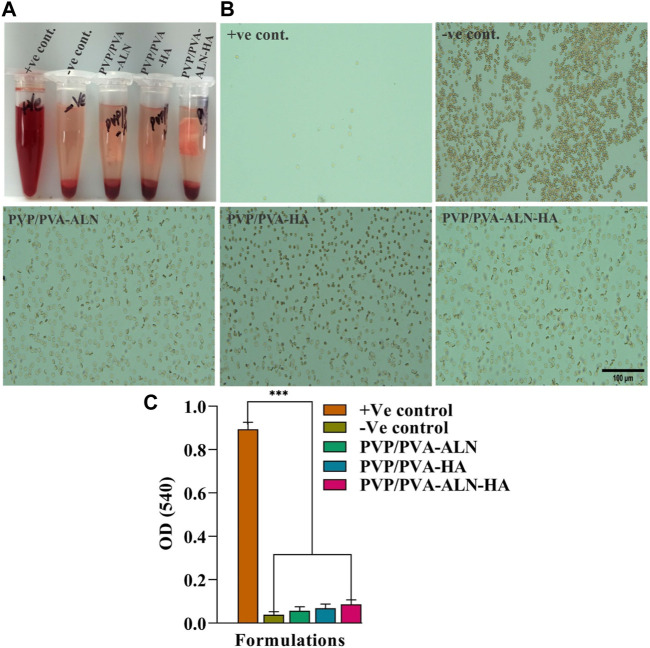
The study of hemolysis and the effect of scaffold on the lysis RBCs; **(A)** The hemolytic behavior of all the groups; **(B)** The microscopic image of intact RBCs status after 3 h of incubation with the control groups and nanofiber group PVP/PVA-ALN, PVP/PVA-HA and PVP/PVA-ALN-HA; **(C)** The histogram demonstrated the absorbance value of +Ve control, -Ve control, PVP/PVA-ALN, PVP/PVA-HA and PVP/PVA-ALN-HA at 540 nm. All the significant values are denoted as *p ≤ 0.05, **p ≤ 0.01, ***p ≤ 0.001.

The non-haemolytic potential of any developed material is the most important characteristic, required for any type of tissue regeneration applications. The scaffolds developed for such type of applications should possess haemolytic index less than 5%. Achieving a hemolytic index lower than 2% would indicate excellent biocompatibility. The hemolytic outcomes for all the groups are illustrated in Figure 5A. Microscopic examination of the obtained images provided valuable insights into the haemolytic behaviour of the different materials (Figure 5B). Remarkably, the results clearly demonstrated that PVP/PVA-ALN, PVP/PVA-HA, and PVP/PVA-ALN-HA, exhibited non-haemolytic characteristics that were notably similar to those observed with the–Ve control, as there was no lysis occurred in these groups. This outcome indicates that the interaction between the composite scaffolds and RBCs did not lead to any significant haemolytic effects. However, in +Ve control, intact RBCs were not observed. The haemolytic % of all the groups compared to + Ve control was less than 2%, indicating its high biocompatibility (Figure 5C). The absence of haemolysis in the nanofiber composite scaffolds suggests their potential suitability for biomedical applications, particularly where blood-contacting materials are concerned. These findings underscore the biocompatible nature of the developed nanofiber scaffolds, enhancing their attractiveness for various regenerative approaches.

### 3.5 *In vitro* biological characterization

#### 3.5.1 *In vitro* cytotoxicity

**FIGURE 6:**
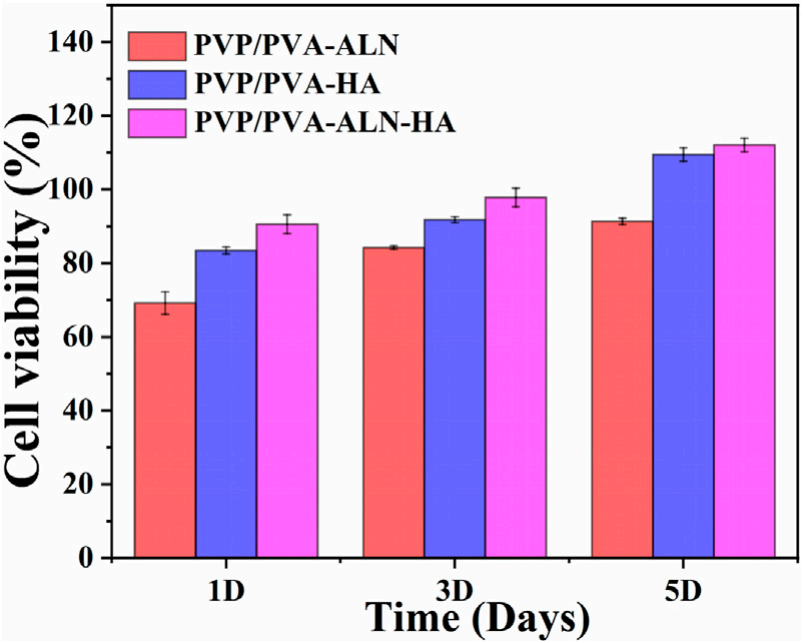
Cell viability study of the nanofibrous scaffold (PVP/PVA-ALN, PVP/PVA-HA and PVP/PVA-ALN-HA) for 1, 3 and 5 days on MC3T3-E1 cells.

Cell viability is intricately linked to the extent of new bone formation, while initial cell adhesion commonly governs cellular function and eventual tissue integration. The presence of greater amounts of bone tissue around the scaffolds can be attributed to the enhanced adhesion and proliferation of stem cells (Wang et al., 2014). To showcase the cell viability of MC3T3-E1 cells on nanofibers *in vitro*, a CCK-8 assay was conducted to determine cell counts after 1, 3, and 5 days. The results reveal that all tested nanofibers (PVP/PVA-ALN, PVP/PVA-HA, and PVP/PVA-ALN-HA) foster the proliferation of MC3T3-E1 cells (Figure 6). Notably, nanofibers containing HA, such as PVP/PVA-HA and PVP/PVA-ALN-HA composite nanofibers, exhibited robust cell viability among MC3T3-E1 cells. This enhanced cell proliferation can be attributed to the synergistic effect of both ALN and HA, which may expedite bone tissue regeneration and wound healing. This notion is grounded in the extensive application of HA in various biotechnological domains, including tissue regeneration, biomedical imaging, bone repair, and drug delivery. Furthermore, HA has demonstrated significant roles in cell proliferation and growth (Januariyasa et al., 2020). The findings indicate that materials with low toxicity can serve as preliminary indicators for the proliferation assessment of MC3T3-E1 cells on these materials. The better biocompatibility of the composite scaffolds, namely, PVP/PVA-ALN, PVP/PVA-HA, and PVP/PVA-ALN-HA scaffolds, is evident from the results. These outcomes signify that the incorporation of an appropriate quantity of ALN and HA nanoparticles into PVP/PVA nanofibers can effectively enhance cell adhesion and proliferation. Figure 6 also showed that embedding HA into electrospun PVP/PVA leads to improved cell viability compared to PVP/PVA-ALN scaffolds. This observation aligns with the established understanding that the addition of HA increases scaffold surface roughness and mechanical strength, while providing functional sites for cell adhesion. Consequently, this study affirms that the fabricated composite nanofiber PVP/PVA-ALN-HA exhibits favorable cytocompatibility with MC3T3-E1 cells.

#### 3.5.2 Live/dead assay

**FIGURE 7:**
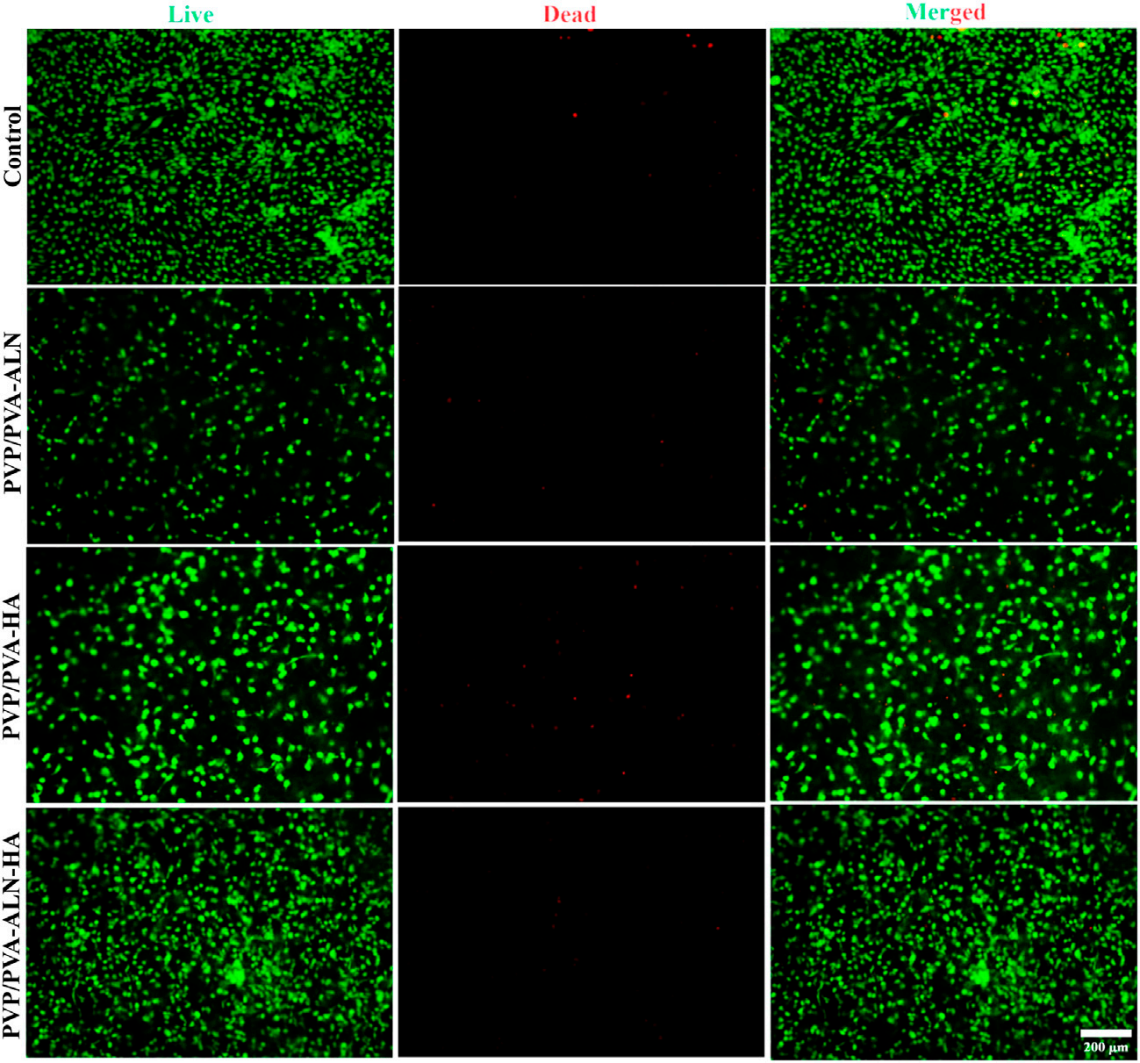
Fluorescence microscopic images of live/dead staining assay of MC3T3-E1 cells cultured with PVP/PVA-ALN, PVP/PVA-HA, PVP/PVA-ALN-HA on nanofiber scaffold surfaces for 3 days.

The cytotoxicity of nanofibers was further confirmed by the results of live/dead staining of MC3T3-E1 cells on the scaffold surfaces, in which the live cells were stained green, while the dead cells were stained red (Figure 7). Dead cells were rarely found in live/dead-stained images, and most of the MC3T3-E1 cells were alive and uniformly distributed, suggesting that all scaffolds had excellent cytocompatibility. Fluorescent images revealed that MC3T3-E1 cells were more spread out on PVP/PVA-ALN-HA scaffold compared to other scaffolds after 3 days in culture. This was consistent with reports that ALN and HA effectively improved the bioactivity of PVP/PVA composites.

#### 3.5.3 Cell adhesion test onto nanofiber scaffolds

**FIGURE 8:**
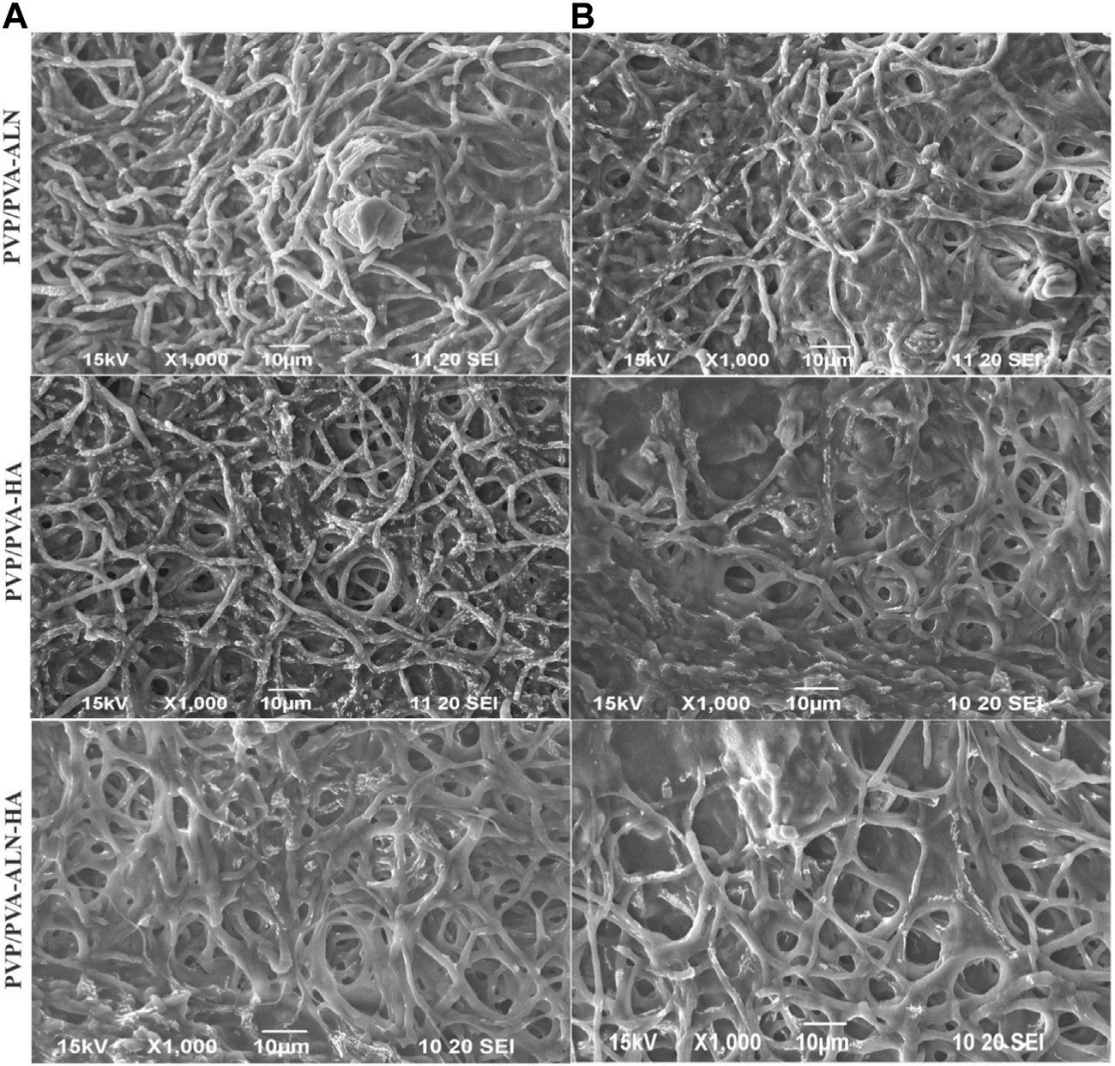
SEM images of MC3T3-E1cells seeded on electrospun nanofiber **(A)** after 1 day presents the adhered cells; **(B)** cell adhesion after 3 days of seeding.

The cellular morphology was visualized using SEM over different time intervals for the respective nanofiber scaffolds. Figure 8 displays the adhesion and proliferation of MC3T3-E1 cells after treatment with the prepared drug-loaded nanofibers for 1 and 3 days. The SEM images reveal that the seeded cells adhered well to the surface of the scaffolds (Figure 8A), underscoring the favourable biocompatibility of the scaffolds. The results indicate that all tested nanofibers facilitated robust cell adhesion, even after prolonged contact between the nanofibers and MC3T3-E1 cells. Notably, PVP/PVA-ALN-HA exhibited the highest cell adhesion and proliferation behaviour. Over time, cell proliferation increased (Figure 8B), and by the third day, the scaffold appeared to be fully covered with highly spread cells, indicating the excellent biocompatibility of the composite nanofibers. The cells have effectively enveloped the nanofibrous scaffold, highlighting the importance of aligning the scaffold’s structure with the extracellular matrix (ECM). These observations strongly suggest that the developed nanofibrous scaffolds hold significant potential for promoting bone tissue regeneration.

**FIGURE 9:**
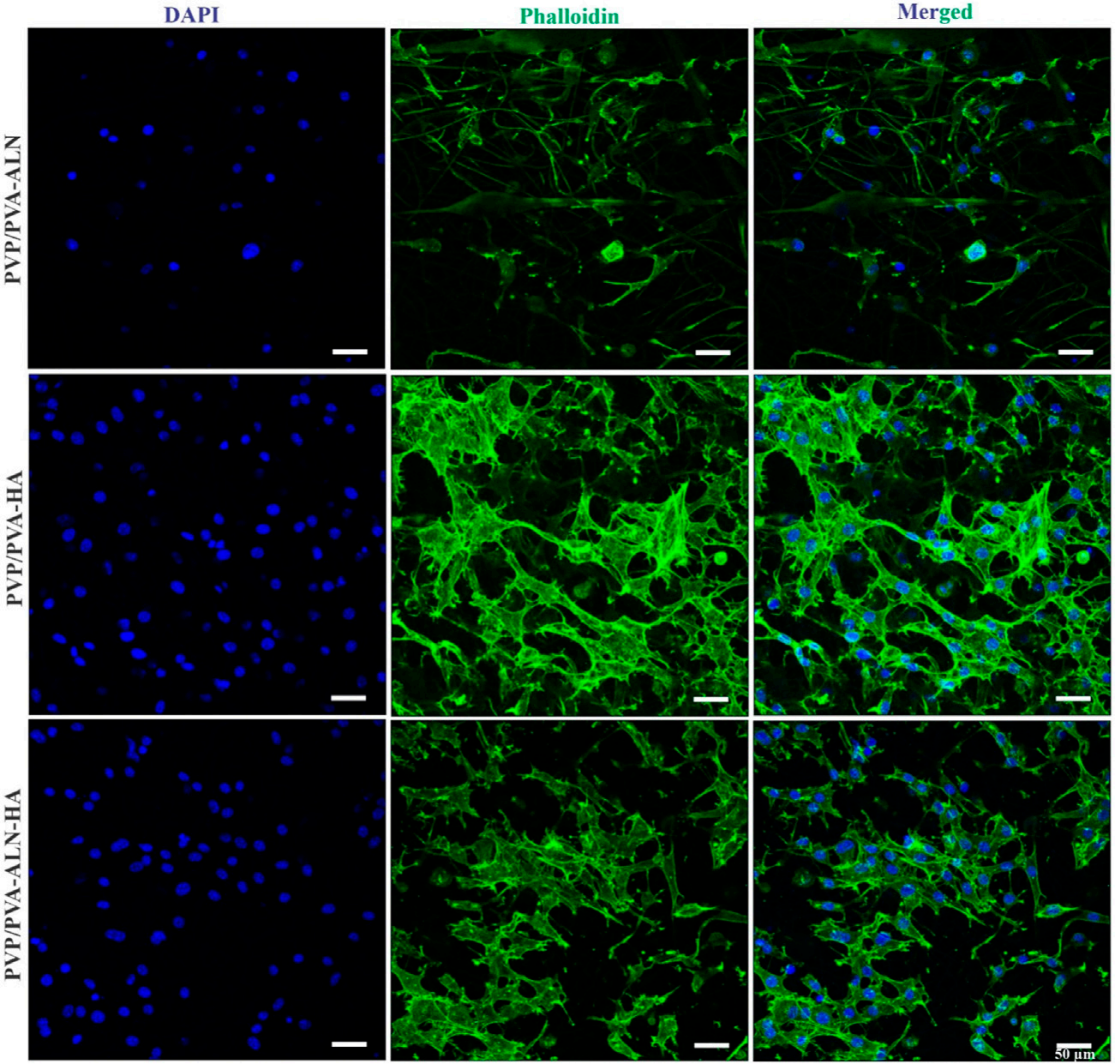
CLSM images of PVP/PVA-ALN, PVP/PVA-HA, PVP/PVA-ALN-HA nanofiber composite scaffolds stained with DAPI and Phalloidin dye on MC3T3-E1cells after 3 days of incubation on scaffolds.

The confocal microscopy images presented in Figure 9, confirmed that PVP/PVA-ALN, PVP/PVA-HA, PVP/PVA-ALN-HA nanofibers composite scaffolds are non-toxic to MC3T3-E1 cells**.** The use of confocal microscopy provided a powerful tool to capture intricate details of the cell on nanofibers. The high-resolution images acquired showcased the cell nuclei stained with DAPI, providing insight into the distribution and adhesion of the MC3T3-E1 cells on the PVP/PVA nanofiber scaffolds. Furthermore, the visualization of the actin cytoskeleton, illuminated by phalloidin staining, confirmed the cell adhesion and proliferation of MC3T3-E1 cells on PVP/PVA-ALN, PVP/PVA-HA, PVP/PVA-ALN-HA. The presence of densely proliferated cells displaying an elongated, spindle-like morphology is particularly noteworthy, as this is a characteristic trait of MC3T3-E1 cell lines. It is evident from Figure 9 that PVP/PVA-HA and PVP/PVA-ALN-HA showed higher cell proliferation as compared to PVP/PVA-ALN group. So, it may be concluded that addition of HA in PVP/PVA nanofiber significantly enhanced the cell growth.

#### 3.5.4 ALP activity measurement

**FIGURE 10:**
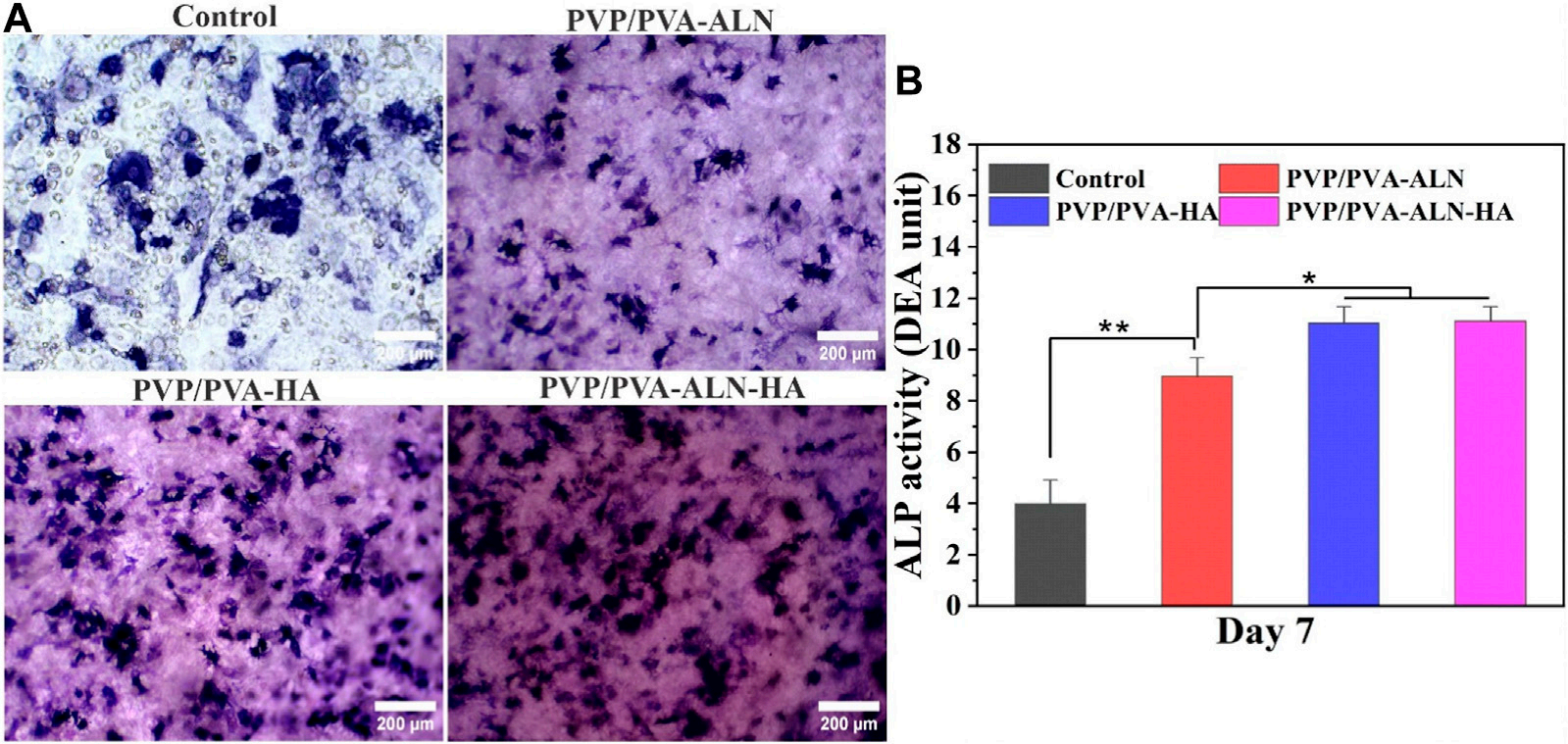
Osteogenic effect of the different PVP/PVA-ALN, PVP/PVA-HA and PVP/PVA-ALN-HA nanofibers, **(A)** ALP staining after 7 days of culture; **(B)** Quantitative analysis of ALP staining showing different groups control, PVP/PVA-ALN, PVP/PVA-HA and PVP/PVA-ALN-HA nanofiber scaffolds. *p*-value < 0.05 and 0.01 were represented by * and **, respectively, considered as significant.

The promotion of osteogenic differentiation around the interface between implant materials and bone is of paramount importance for successful bone reconstruction. An ideal bone implant should not only demonstrate favourable cytocompatibility but also encourage osteogenic activity (Wang and Lu, 2020). Building upon the positive results obtained from *in vitro* cell line studies, the early osteogenic differentiation of MC3T3-E1 cells induced by different scaffolds was confirmed through the assessment of ALP activity. ALP activity serves as a biochemical marker for osteoblastic activity, manifesting an increase in the early stages of osteogenesis and signifying differentiation towards osteoblasts (Gouma et al., 2012). The staining density and distribution of ALP in MC3T3-E1 cells cultured on PVP/PVA-ALN, PVP/PVA-HA, and PVP/PVA-ALN-HA were found to be higher than the control group, as illustrated in Figure 10A. Quantitative analysis further corroborated the staining results (Figure 10B). Notably, there was no significant difference observed between PVP/PVA-HA and PVP/PVA-ALN-HA nanofibers. This suggests that the presence of HA in the scaffold promoted osteogenic differentiation, thereby facilitating the development of new bone tissue. The ability of HA to stimulate osteogenic differentiation of stem cells has been supported by numerous studies (Behere et al., 2021; Niu et al., 2021). However, the combination of ALN and HA exhibited additive effects on osteogenesis. By incorporating ALN within a safe range and ensuring controlled release via a dual delivery system, the potential side effects of ALN can be mitigated. This approach aligns with an appropriate strategy to harness the benefits of ALN without undesirable consequences. These findings are consistent with previous research (Park et al., 2015; van Houdt et al., 2018). Overall, the prepared PVP/PVA-ALN-HA nanofiber scaffold demonstrated promising capabilities for osteogenic differentiation, rendering it feasible for application in bone regeneration scenarios.

#### 3.5.5 Osteoclast formation

Bone healing is a multifaceted process that involves not only osteoblasts but also osteoclasts. Effective reduction in osteoclastogenesis can offer advantages for successful bone regeneration. Osteoclasts, responsible for bone mineral resorption, are formed through the fusion of hematopoietic cells of the monocyte-macrophage lineage, induced by specific stimuli like receptor activator of nuclear factor κB ligand (RANKL) and macrophage colony-stimulating factor (M-CSF) (Lee et al., 2018). The development of multinucleated osteoclasts is governed by the activation of intracellular pathways, including nuclear factor κB (NF-κB) and nuclear factor of activated T cells c1 (NFATc1) (Ahmad et al., 2020). Studies have suggested that ALN can inhibit osteoclast formation by suppressing the activation of the ERK1/2 and Akt pathways (Jiang, Mao, and Gao, 2015).

**FIGURE 11:**
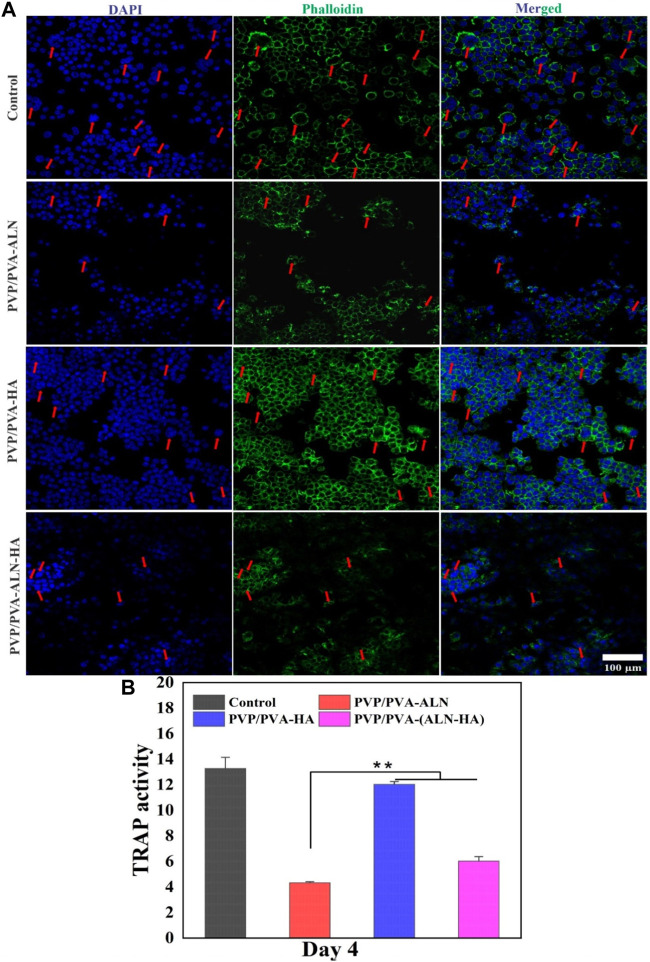
**(A)** Representative nucleus/cytoskeleton staining images of RAW 264.7 cells grown on different PVP/PVA-ALN, PVP/PVA-HA and PVP/PVA-ALN-HA nanofiber composite scaffolds whereas cytoskeleton and cell nuclei were stained by Phalloidin (green) and DAPI (blue), respectively (red arrows represent the formed osteoclasts); **(B)** Enzyme activity of TRAP from RAW 264.7 cells cultured on different scaffolds. *p*-value < 0.05 and 0.01 were denoted by * and **, respectively.

To assess the impact of the nanofibers on osteoclastic activity, the morphology of osteoclast differentiation was examined. In Figure 11A, numerous large multinucleated osteoclasts were observed on the surface of the PVP/PVA-HA scaffold and the control group. In contrast, significantly fewer multinucleated osteoclasts were present in the PVP/PVA-ALN and PVP/PVA-ALN-HA groups. The development and maturation of functionally active osteoclasts encompass several complex steps, including precursor cell fusion to form multinucleated cells, significant cytoskeletal rearrangements, adhesion, polarization, and actin ring formation.

The effect of ALN-containing scaffolds on osteoclasts was further evaluated through TRAP activity, an enzyme marker of osteoclasts (Huang et al., 2019). As shown in Figure 11B, TRAP activity of RAW 264.7 cells in the ALN-loaded nanofiber groups exhibited a significant reduction (*p* < 0.01) compared to other groups. This suggests that ALN played a role in inhibiting osteoclast maturation. Therefore, the findings highlight that ALN-HA loaded nanofibers have the potential to inhibit osteoclast formation, indicating their potential role in promoting bone formation. This bodes well for their application in bone regeneration scenarios.

## 4 Conclusion

The objective of this study was to develop nanofiber scaffolds that could effectively balance bone remodelling and regeneration processes. Through the electrospinning technique, we successfully created nanofibers loaded with ALN and HA nanoparticles. These nanoparticles were seamlessly integrated into a PVP/PVA nanofiber scaffolds to enhance its overall properties. The combination of ALN and HA within the electrospun PVP/PVA-ALN-HA nanofibers aimed to synergistically regulate the equilibrium between bone resorption and formation. Our findings demonstrated that the nanofibers possessed a porous and highly hydrophilic network structure, resembling the natural ECM of human bone tissue. Incorporating ALN and HA into the nanofibers led to increased water retention capacity and desired biodegradability for bone regeneration. Furthermore, HA incorporation improved the mechanical strength and thermal stability of PVP/PVA-ALN-HA. EDX analysis confirmed the successful integration of ALN and HA nanoparticles into the PVP/PVA nanofibers. Notably, PVP/PVA-ALN-HA nanofibers exhibited a sustained release of ALN over 2 weeks.

*In vitro* evaluations further corroborated the biocompatibility of the scaffolds, demonstrating non-toxicity and supporting bone cell adhesion and proliferation on MC3T3-E1 cells, evidenced by SEM images after 1 and 3 days of incubation. The cell adhesion and proliferation of scaffolds was further assessed by CLSM images on MC3T3-E1 cells after 3 days of incubation. The fluorescence microscopy images of Live/dead assay revealed the absence of dead cells. Significantly higher level of ALP in PVP/PVA-HA and PVP/PVA-ALN-HA groups as compared to control, promoted osteogenic differentiation which leads to formation of new bone tissue. Moreover, our studies indicated that the PVP/PVA-ALN-HA nanofiber scaffold had the potential to enhance osteogenesis and significantly inhibited the multinucleated osteoclast formation. However, the reduction in TRAP level in ALN containing nanofiber scaffolds confirmed the inhibition of osteoclast formation. These collective results indicate that the fabricated PVP/PVA-ALN-HA nanofibrous scaffolds could serve as a promising platform for bone regeneration applications and this suggest the potential consideration of *in-vivo* studies in future.

## Data Availability

The original contributions presented in the study are included in the article/supplementary material, further inquiries can be directed to the corresponding authors.
